# Influence of Magnesium Alloy Degradation on Undifferentiated Human Cells

**DOI:** 10.1371/journal.pone.0142117

**Published:** 2015-11-23

**Authors:** Francesca Cecchinato, Nezha Ahmad Agha, Adela Helvia Martinez-Sanchez, Berengere Julie Christine Luthringer, Frank Feyerabend, Ryo Jimbo, Regine Willumeit-Römer, Ann Wennerberg

**Affiliations:** 1 Department of Prosthodontics, Faculty of Odontology, Malmö, Sweden; 2 Helmholtz-Zentrum Geesthacht, Geesthacht, Germany; North Carolina A&T State University, UNITED STATES

## Abstract

**Background:**

Magnesium alloys are of particular interest in medical science since they provide compatible mechanical properties with those of the cortical bone and, depending on the alloying elements, they have the capability to tailor the degradation rate in physiological conditions, providing alternative bioresorbable materials for bone applications. The present study investigates the *in vitro* short-term response of human undifferentiated cells on three magnesium alloys and high-purity magnesium (Mg).

**Materials and Methods:**

The degradation parameters of magnesium-silver (Mg2Ag), magnesium-gadolinium (Mg10Gd) and magnesium-rare-earth (Mg4Y3RE) alloys were analysed after 1, 2, and 3 days of incubation in cell culture medium under cell culture condition. Changes in cell viability and cell adhesion were evaluated by culturing human umbilical cord perivascular cells on corroded Mg materials to examine how the degradation influences the cellular development.

**Results and Conclusions:**

The pH and osmolality of the medium increased with increasing degradation rate and it was found to be most pronounced for Mg4Y3RE alloy. The biological observations showed that HUCPV exhibited a more homogeneous cell growth on Mg alloys compared to high-purity Mg, where they showed a clustered morphology. Moreover, cells exhibited a slightly higher density on Mg2Ag and Mg10Gd in comparison to Mg4Y3RE, due to the lower alkalinisation and osmolality of the incubation medium. However, cells grown on Mg10Gd and Mg4Y3RE generated more developed and healthy cellular structures that allowed them to better adhere to the surface. This can be attributable to a more stable and homogeneous degradation of the outer surface with respect to the incubation time.

## Introduction

Clinical modalities for orthopaedic trauma require the use of non-resorbable screws, plates, stents and pins made of metallic materials such as titanium, cobalt-chrome and stainless steel alloys [1–3]. However, the major disadvantage of these materials is that, in some cases, it will be necessary for the clinicians to remove the device at a certain time of recovery. Due to this drawback, the constant pursuit for alternative bioresorbable materials that could function as orthopaedic and oromaxillofacial applications has been increased. Magnesium and magnesium alloys have drawn significant attention due to their biodegradable characteristics [4–6]. These materials combine the resorbable properties of the polymeric implants which are widely used for osteosynthesis in non-weight bearing bones [7], with the mechanical stability of metal implants, which withstand the mechanical loading during function [8]. Although these degradable materials are promising, the most challenging issue of using magnesium-based materials is controlling their degradation behaviour in aqueous environments, that is accompanied by hydrogen gas evolution and chemical surface alteration, which does not properly match the bone healing rate *in vivo* [9–12].

Initial cell adhesion and spreading immediately after implant insertion into the host tissue are essential biological processes for establishing connections between cells and giving a stable crosslink for the upcoming cellular events around the implant surface [13, 14]. It has been proven that an alkaline and hypertonic environment negatively affects cells growth, counteracting initial proliferation and subsequent tissue formation [15, 16]. Under cell culture conditions, it is well known that the chemical formation of favourable degradation products, such as magnesium carbonate (MgCO_3_) and magnesium hydroxide (Mg(OH)_2_) as well as their solubility is pH-dependent. For example, in a pH range between 7.5–8.5 (which is the standard setup for the *in vitro* experiments), both MgCO_3_ and Mg(OH)_2,_ tend to partly dissolve, inducing an alkalinisation effect of the surrounding environment [9, 17]. Therefore, magnesium degradation has a direct influence on cell adhesion and proliferation, as its degradation is accompanied with hydrogen evolution and hence environment alkalinisation. Further questionable influence is, whether the topographical features and chemical composition of the degraded surface can influence cell adhesion and development on implant surface.

The bone-magnesium implant interaction has been investigated with regards to Mg4Y3RE, a commercially available magnesium alloy which shows a promising potential in bone mass generation and mineralization *in vivo* compared to a degrading polymer [11, 18, 19]. It has also been reported that Mg4Y3RE alloy presents good degradation behaviour under *in vitro* conditions [20, 21]. However, the initial degradation rate is too high and localized at the peri-implant region *in vivo* [11]. Mg-Ag alloys reported excellent mechanical properties and a slow degradation rate in vitro and also the promising antibacterial effect of Ag ions [22, 23]. Magnesium alloys containing low percentages of gadolinium (Gd) have been produced and characterised and the outcomes suggested that this alloying element in specific concentration slowed down the degradation process and improved the mechanical properties of the alloy [5]. Even though Gd toxicity is a concern, reported data on its cytotoxicity have demonstrated that the toxic effect is within the applicable level and that Mg-Gd alloy can be considered as a good candidate for medical applications [24–27].

In the present study, the degradation properties of Mg2Ag, Mg10Gd and Mg4Y3RE alloys were analysed under cell culture conditions in comparison to high-pure magnesium Mg. Mesenchymal stem cells are the first cells that migrate and adhere at the peri-implant site, therefore human umbilical cord perivascular cells (HUCPV) were used in this study since they possess pluripotent plasticity and high proliferation rate. HUCPV viability and adhesion structures were examined with staining techniques: LIVE/DEAD staining assay and immunocytochemical assay, respectively. It was hypothesized that the degradation behaviour and surface chemical as well as composition of the three Mg alloys differently influenced early cell viability and adhesion *in vitro*, providing a preliminary indication of the suitability of these alloys as biodegradable metals to proceed *in vivo*.

## Experimental Procedures

### Materials production

The following materials were used to produce alloys for this study: magnesium (99,99%, Xinxiang Jiuli Magnesium co. ltd, China), yttrium (Y; 99,95%, Grirem Advanced Materials Co. Ltd., China), Gadolinium (Gd; 99,95%, Grirem Advanced Materials Co. Ltd., China), rare earth mixture (RE; Grirem Advanced Materials Co. Ltd., China), and silver (Ag; 99.99%, ESG Edelmetall-Handel GmbH & Co. KG, Germany). Three magnesium-based materials were produced: Mg2Ag (1.89% Ag, the rest was Mg), Mg10Gd (8.4% Gd, the rest was Mg), and Mg4Y3RE (3.45% Y, 2.03% Nd, 0.84% Ce, the rest was Mg). High-purity magnesium (99.97% Mg) was used as a control. The concentration of Mg, Y, Nd and Ce in Mg4Y3RE were determined by spark emission spectrometry (Spectrolab M, Spektro, Germany) and the concentration of Ag in Mg2Ag and Gd in Mg10Gd were determined by X-ray fluorescence spectrometry (Bruker AXS S4 Explorer, Bruker AXS GmbH., Germany). The materials were cast at HZG-MagIC.

The three magnesium alloys (Mg2Ag, Mg10Gd, and Mg4Y3RE) were produced by permanent mould gravity casting. After melting of pure Mg, the melt was hold at 720°C and the preheated alloying elements were added with continuous stirring for 15 min. The melt was poured into a pre-heated (550°C) permanent steel mould treated with boron nitride. During the casting process cover gas was used (SF_6_ and Ar mixture). The alloys were homogenized with a T4 heat treatment prior to extrusion in Ar atmosphere at 550°C (Mg10Gd and Mg4Y3RE) and at 420°C (Mg2Ag) for 6 h. Afterwards, the alloys were extruded indirectly with an extrusion ratio 4/25 (Strangpreßzentrum Berlin, Berlin, Germany). The chamber of the extrusion machine was set to 370°C and the billets (d = 30mm) were pre-heated for 1 h at 370°C (Mg2Ag), at 390°C (Mg4Y3RE) and at 430°C (Mg10Gd). The extrusion speed was between 3 and 4.5 mm/sec. High-pure Mg was cast by permanent mould direct chill cast. The cast billet (d = 110mm) was extruded indirectly with an extrusion ratio 1/84. The temperature of billet was 340°C and the speed of the extrusion was 0.7 mm/sec. Discs (diameter of 10 mm and 1.5 mm thickness) were machined from the extruded bars, surface finishing was performed by lapping (Henschel KG, Munich, Germany). Sterilization was performed with gamma irradiation at a total dose of 27 kGy (BBF Sterilisationsservice, Kersen, Germany).

### Sample pre-incubation

Samples were pre-incubated for 24, 48 and 72 h in order to evaluate how cells behave and adhere onto different degradation states of the materials. Samples were placed in 12-well plates, one sample per well, according to the experimental layouts. The extraction volume has been calculated according to the guidelines in ISO 10993–12 [28]: 3 mL of modified eagle medium (alpha MEM, Life Technologies, Gibco) supplemented with 15% foetal bovine serum (FBS, Life Technologies, Gibco) and 1% penicillin/streptomycin (p/s) were added to each sample and incubated for 24 h, 48 h and 72 h under cell culture conditions (37°C, 5% CO_2_ and 95% controlled humidity atmosphere).

### In vitro degradation parameters

#### Determination of degradation rate, pH and osmolality

The pH (Sentron Argus X pH-meter, Fisher Scientific GMBH, Schwerte, Germany) and the osmolality (Osmomat 030, Gonotec, Berlin, Germany) of the immersion medium were measured before and after 24 h, 48 h and 72 h of pre-incubation. For each pre-incubation time 5 samples of each alloy were immersed in the immersion medium (α-MEM + 15% FBS + 1% p/s) under cell culture conditions. After immersion, the degradation products were removed by soaking samples in chromic acid (180 g/L in distilled water, VWR international, Darmstadt, Germany) for 20 min at room temperature. The degradation rate (DR) was calculated in unit mm/year by the following equation:

$$DR=\frac{8.76\times \Delta g\times 10^{4}}{A\times t\times \times \rho }$$ (1)

where Δg: weight change (g), A: sample surface area (cm^2^), t: immersion time (hours), ρ: density (g/cm^3^)

#### Energy dispersive X-ray spectroscopy (EDX) with low accelerating voltage

The degraded surfaces and their chemical composition were investigated. Samples were immersed in α-MEM + 15% FBS + 1% p/s for the longest immersion time (72 h) to analyse possible differences in elements composition between the different alloys. Samples were then dried for 24 h in vacuum and analysed by energy dispersive X-ray spectroscopy (EDX; Apollo XP, EDAX, Ametek GmbH, Wiesbaden, Germany) equipped to a scanning electron microscope (SEM; Auriga, Zeiss, Oberkochen, Germany). Surface measurements were done by accelerating voltage of 10 keV with the secondary electron detector (SE2) aiming to analyse the corroded surface and avoid the high contribution of magnesium in the bulk. Points of interests on the surface were analysed as well. The measurements were done semi-quantitatively without standard sample. EDX quantification was realized on all samples with eight common elements of interest; Mg, O, P, C have been considered despite of the alloying elements in each alloy. To obtain a high counting rate by EDX measurements the SEM aperture of 120μm diameter had been used in High Current mode.

#### Atomic force microscopy (AFM) for surface topography

The surface topography was investigated with atomic force microscopy (AFM, XE-100, Park Systems Corp, Suwon, Korea). Images were obtained at 10×10 μm and were subjected to levelling and Gaussian filtering with a cut-off at 2.5 μm, using the software MontainsMap 6 (Digital Surf, Besançon, France). Results were analysed collecting the following 3-dimensional parameters to describe the surface topography of a biomaterial [29]: the arithmetic mean of the height deviation from the mean plane (S_a_); the number of peaks per unit of area (S_ds_); the ratio between the developed surface area and a flat reference area (S_dr_). The mean value and standard error of the parameters were obtained from 9 scans of each group for each pre-incubation time (a total of 36 measurements), from random sites on the surface.

### In vitro HUCPV cell responses

#### Isolation and culture of human umbilical cord perivascular cells (HUCPV)

HUCPV were isolated with the approval from the local ethical committee Ethik-Kommission der Ärztekammer Hamburg (Hamburg, Germany), following the protocols from Sarugaser et al. [30]. HUCPV were obtained from umbilical cord samples. Written informed consent from the donor was obtained for the use of these samples in research. The cord was cut into pieces of about 5 cm. The vessels were then isolated and tied together at the ends, leading to a vessel loop. Afterwards, they were placed in T-175 cell culture flask and cultured for 10 days in α-MEM and 15% FBS and 1% antibiotics. After outgrowth of cells from the tissues, the medium was changed every 2–3 days. At 80% confluency, the cells were detached and resuspended in fresh medium. For each assay, discs were transferred into agarose pre-coated 12-well plate after each pre-incubation time. Afterwards 50.000 cells in 50 μL were added onto each sample and incubated for 30 min to allow early cell adhesion. Then 3 mL of fresh medium were added and cells were further cultured for 24 h on each pre-incubated sample for each experimental analysis.

#### In vitro cytotoxicity staining assay

*In vitro* qualitative and quantitative analysis of material cytotoxicity was performed by using LIVE/DEAD (Life technologies, Darmstadt, Germany) assay. After samples pre-incubation for 24 h, 48 h and 72 h in cell culture medium and cell culture incubator, 50.000 cells were seeded onto each disc surface and cultured for 24 h. The staining solution was prepared by adding 4 μl Calcein AM (LIVE), and 10 μl Ethidium homodimer-1 (DEAD) to 10 ml of phosphate-buffered saline (PBS). The samples were first washed with PBS to eliminate non-adherent cells, followed by immersion of each sample in 1.5 ml of staining solution, and incubating them under cell culture conditions. The staining solution was then replaced by α-MEM and samples were visualized by fluorescent microscope (Nikon GmbH, Düsseldorf, Germany), the applied filters were Fluoresceine isothiocyanate conjugated (FITC) (Ex: 460–500 nm; Em: 510–560 nm; Mirror at 505 nm) and Texas red (Ex: 540-580nm; Em: 600–660; Mirror at 595 nm). Images were taken in large-image mode with 20x magnification. Afterwards, the intensity profiles of FITC (green) and Texas red (red) were quantified from images taken from 3 samples for each material at each pre-incubation time. The intensity was quantified with microscope imaging software NIS-Elements AR (Nikon GmbH, Düsseldorf, Germany). The results were normalized according to the formula:

$$\mathrm{Viability}\;(\%)=\frac{(\frac{\mathrm{live cells}}{\mathrm{live cells}+\mathrm{dead cells}})\mathrm{sample}}{(\frac{\mathrm{live cells}}{\mathrm{live cells}+\mathrm{dead cells}})\mathrm{control}}\times 100$$

#### Immunocytochemical test for adherent cells

After 24 h of cell culture, samples were carefully transferred to a new 24-well plate and stained according to the manufacturer´s protocol (Actin Cytoskeleton and Focal Adhesion Staining Kit, FAK100, Millipore, Ontario, Canada). 1 mL of fixative (4% paraformaldehyde in 1 x PBS) was added to each sample (two samples per alloy per pre-incubation time) and incubated for 20 minutes at room temperature. Afterwards, discs were washed twice with wash buffer (1xPBS + 0.05% tween-20) and were permeabilized with 1mL permeabilizing reagent (0.15% triton X-100 in 1xPBS) for 5 min at room temperature. 1 mL of blocking solution formed by 1% bovine serum albumin (BSA) in 1 x PBS was added to each sample for 30 min. The first staining was vinculin monoclonal antibody, purified clone 7F9 at 1 mg/mL and diluted 1:250 with the blocking solution. This monoclonal antibody is very specific for the staining of focal contacts in cells (in green). Samples were stained with 1 mL of this working solution for 1h at room temperature. The secondary antibody (Goat anti-Mouse IgG, (H+L) FITC, Millipore) was diluted 1:500 in 1xPBS just before use and samples were incubated with 1 mL of the working solution for 1 h at room temperature. 1 mL of tetramethilrhodamine (TRITC)-conjugated phalloidin was incubated simultaneously with the secondary antibody for double labelling. These immunocytchemical stainings were able to bind the actin filaments within cells (red). In order to visualise the nuclei, counterstaining was performed by incubating cells with 4,6-diamino-2-phenylindole (DAPI), blue, (0.1 mg/mL diluted 1:500 in PBS) for 5 min at room temperature. Three washes with wash buffer were accurately done after each staining steps. Fluorescence images were obtained with a fluorescent microscope (Nikon GmbH, Düsseldorf, Germany) using microscope imaging software NIS-Elements AR (Nikon GmbH, Düsseldorf, Germany).

### Statistical analysis

Statistics were performed on degradation data by SigmaStat package (Systat software GmbH, Erkrath, Germany; version 11.0). Standard analysis comparing more than two conditions was done by using one-way analysis of variance (ANOVA) on ranks with Dunn’s multiple comparison post hoc test. Statistical values are referred to the relevant measurements. The mean values and standard errors from AFM analysis were compared by applying the non-parametric Kruskal-Wallis test, followed by pairwise comparisons with the Mann-Whitney U test (SPSS software, Inc., Chicago. IL, USA). The mean values obtained from the LIVE/DEAD staining intensity quantification were compared with one-way analysis of variance (ANOVA) followed by post-hoc Tukey test for multiple comparisons (OriginLab Origin 9.0 software, MA, USA).

## Results

### In vitro degradation parameters

#### Degradation rate, pH and osmolality

**Fig 1:**
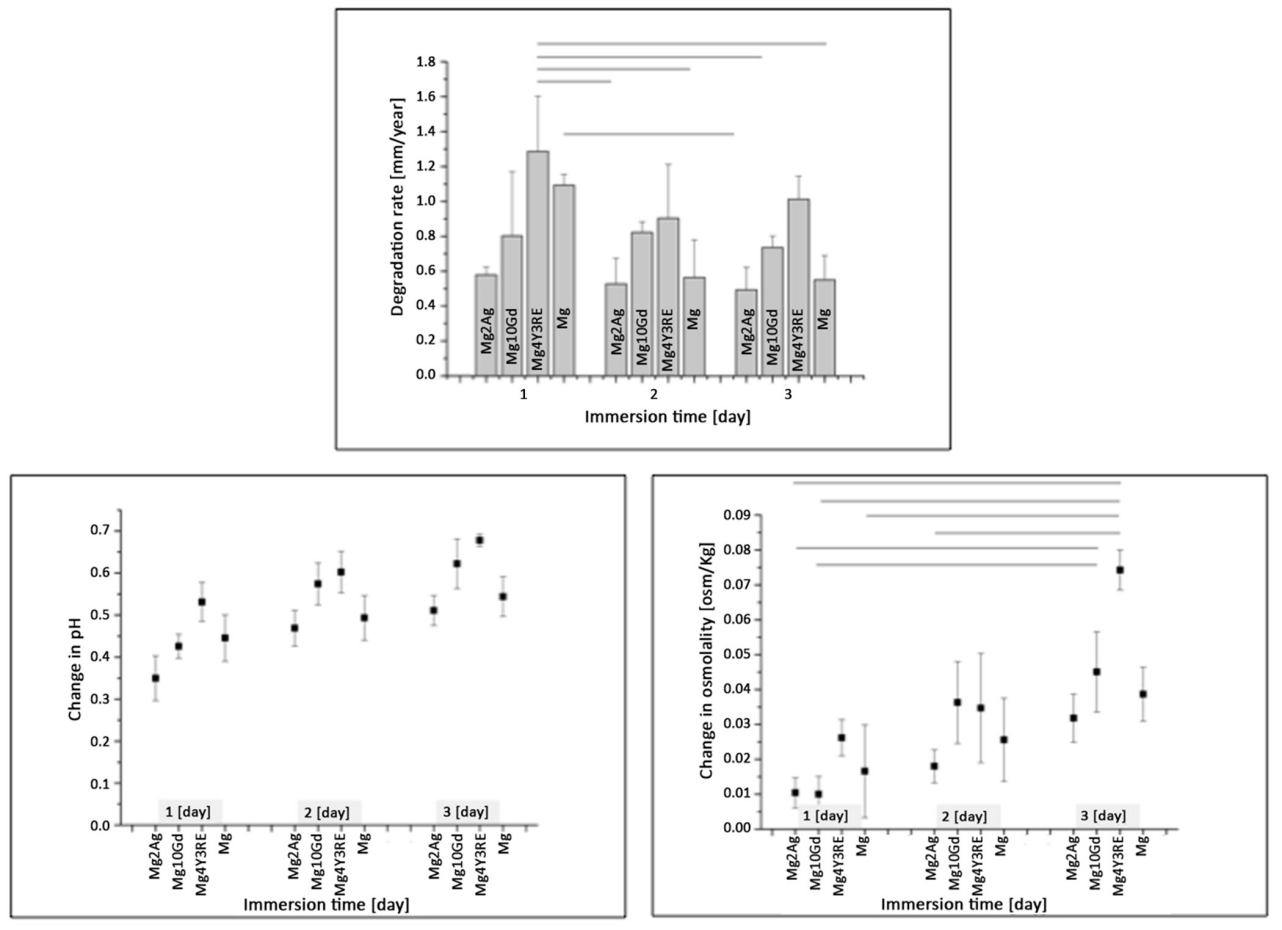
Degradation rate [mm/year], pH and osmolality [osm/kg] after immersion for 24 h, 48 h, and 72 h in corrosion medium under cell culture conditions. The basic solution is α-MEM with addition of 15% FBS and 1% p/s. Significant differences in groups are marked by lines (significance level p < 0.05; n = 5 per group).

The calculated degradation rate for the different alloys as well as pH and osmolality of the immersion media after three pre-incubation times can be seen in Fig 1. Comparing the degradation rate for each alloy at different points time and between the studied alloys, Mg4Y3RE and high-pure Mg show high degradation rates during the first 24 h, which is correlated to the higher increase in pH and osmolality. The high initial interaction between the material and the surrounding environment is reduced after 48 h, leading to a reduction in degradation rate by >50% for the pure Mg samples. Mg10Gd shows a stable degradation over time with moderate increase of pH and osmolality. This behaviour is observed also for Mg2Ag samples but with lower values and lower effects on the surrounding environment.

#### Surface chemical analysis

**Fig 2:**
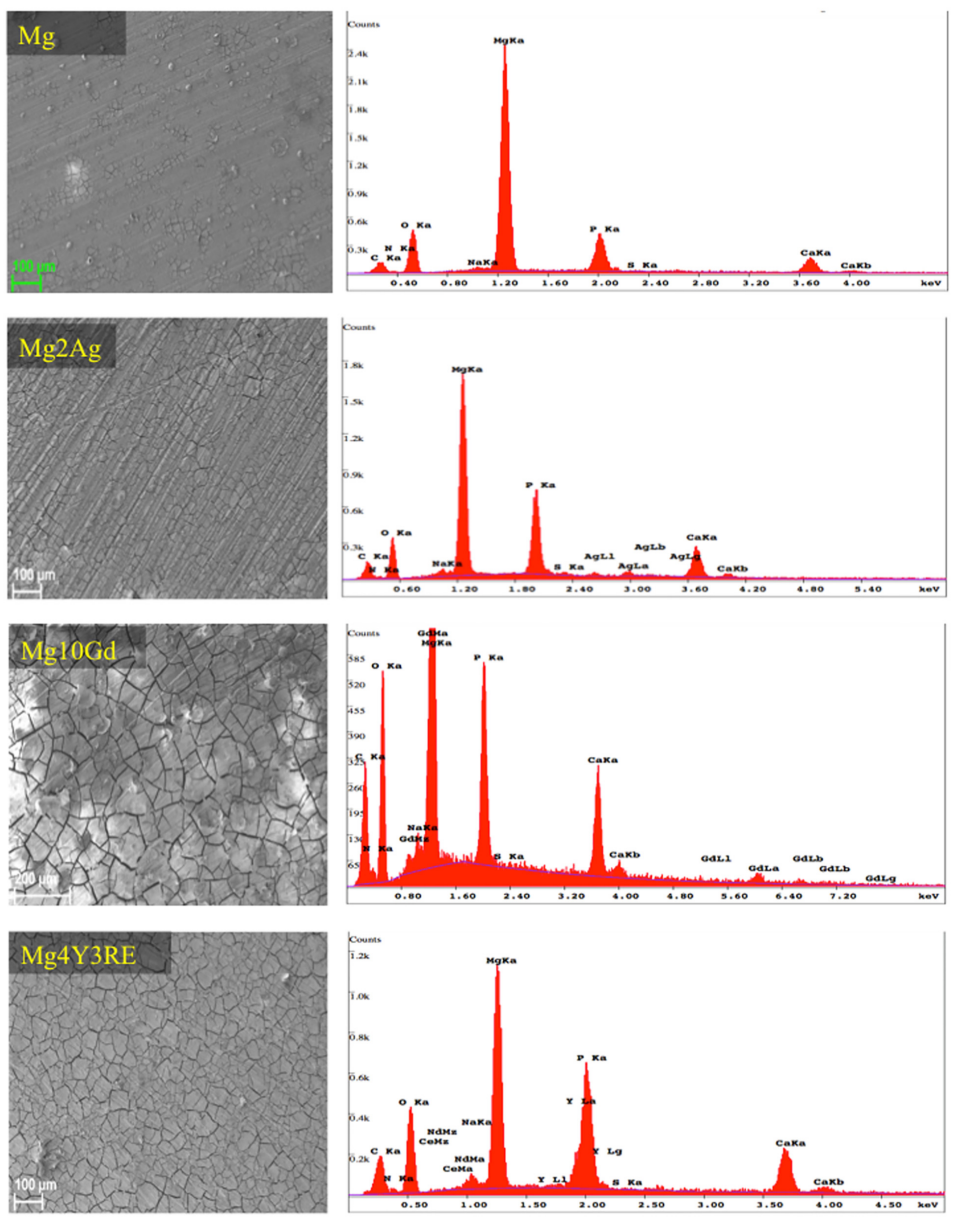
SEM images and the corresponding EDX analysis of magnesium alloys and hp Mg. Analysis was performed after 72 h pre-incubation time applying 10 KeV accelerating voltage.

SEM images after 72 h as pre-incubation time procedure show spherically shapes on the surface of all alloys (Fig 2). Element weight % quantified out of these points shares the same trend in concentrations, basically high oxygen (O) concentration with carbon (C), Mg and phosphorous (P) contributions.

EDX area measurements with an energy of 10 keV were performed on each materarial suface. To have a rough idea about the most abundant elements on the different degraded alloys surface, weight % of interesting elements are presented in Table 1. Results show that Mg2Ag differs from the other alloys in particully high P content. Whereas Mg10Gd and Mg4Y3RE show comparable element distribution on surface with high carbon contribution in comparision to pure Mg and Mg2Ag.

**Table 1:** Weight % of O, C, P and Mg quantified out of the EDX mapping with 10 keV on Mg, Mg2Ag, Mg10Gd and Mg4Y3Re after 72 h of pre-incubation in culture medium and in cell culture conditions.

	O (%)	C (%)	P (%)	Mg (%)
**Mg**	85.1	2.1	2.4	9.9
**Mg2Ag**	75.3	1.87	10.6	11
**Mg10Gd**	88.1	5.8	1.38	3.43
**Mg4Y3RE**	89.1	4.71	1.72	3.33

#### Surface nanotopography

**Fig 3:**
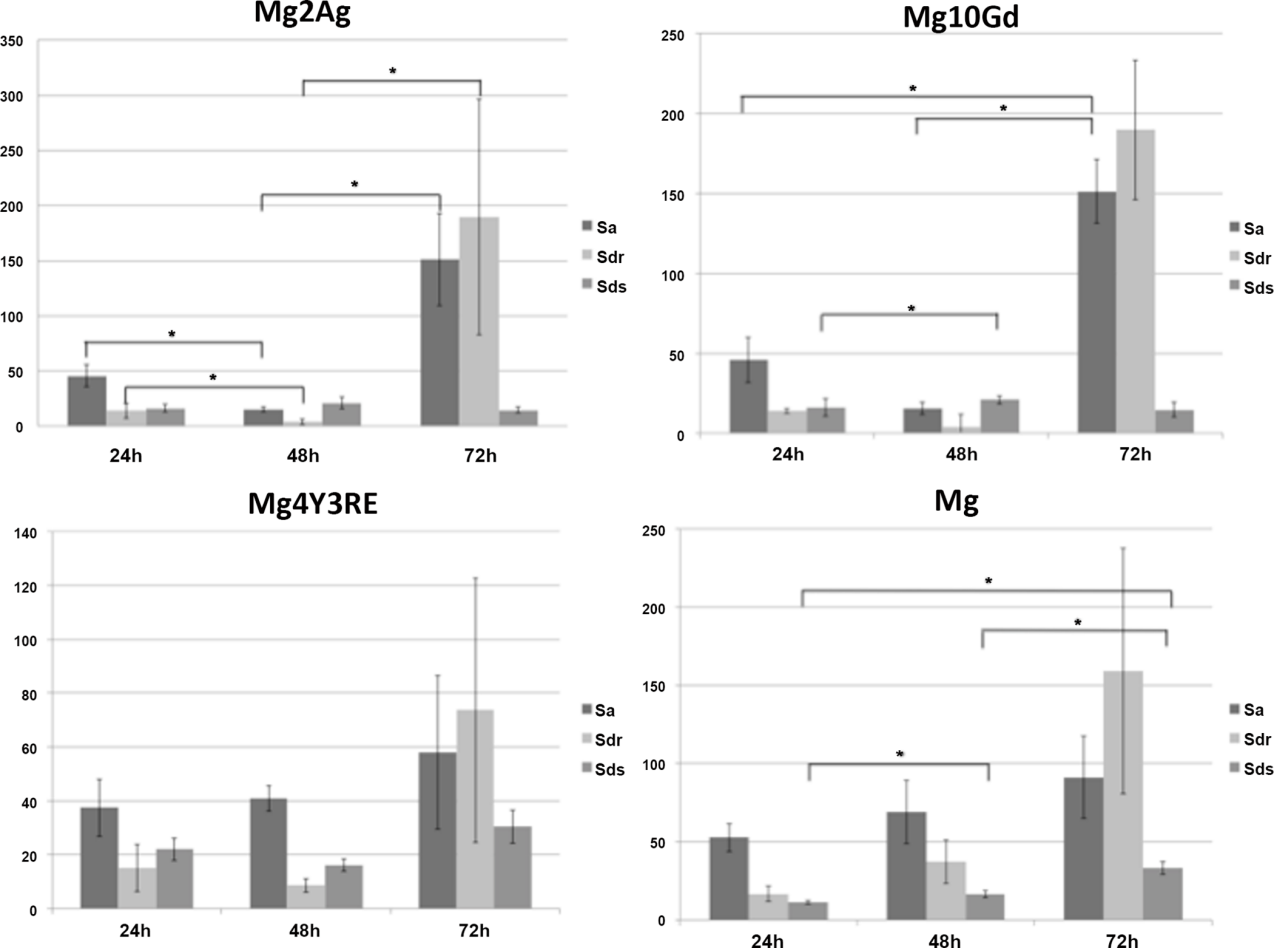
Mean ± standard error for roughness parameters measured with AFM at 10x10 μm for Mg2Ag, Mg10Gd, Mg4Y3RE and Mg after 24, 48 and 72 hours of pre-incubation; p-values for Kruskal-Wallis followed by pairwise comparisons using the Mann-Whitney test. Significant differences are marked by an asterisk (significance level *p* < 0.05).

The surface roughness was analysed with AFM, describing it through S_a_, S_dr_ and S_ds_ dimensional parameters. From the statistical analysis it was observed that the surface nanotopography significantly changed during the degradation process. Fig 3 shows that the overall trend was characterized by a decrease in surface roughness for Mg2Ag and Mg10Gd between 24 h and 48 h, followed by a significant increase with a further 24 h of degradation. Particularly, Mg2Ag surface was more affected by initial degradation, significantly reducing average roughness (S_a_) and specific surface area (S_dr_) between 24 h and 48 h of degradation. Conversely, it can be observed that Mg4Y3RE as well as high-pure Mg did not show significant increase in S_a_ even though it followed the same surface degradation trend of the other two alloys.

### In vitro HUCPV cell responses

The following paragraphs show the correlation between cell behaviour (24 h of culture) and Mg alloys with respect to the degradation states, comparing them with Mg.

#### Cytotoxicity of Mg-based alloys in different degradation states

**Fig 4:**
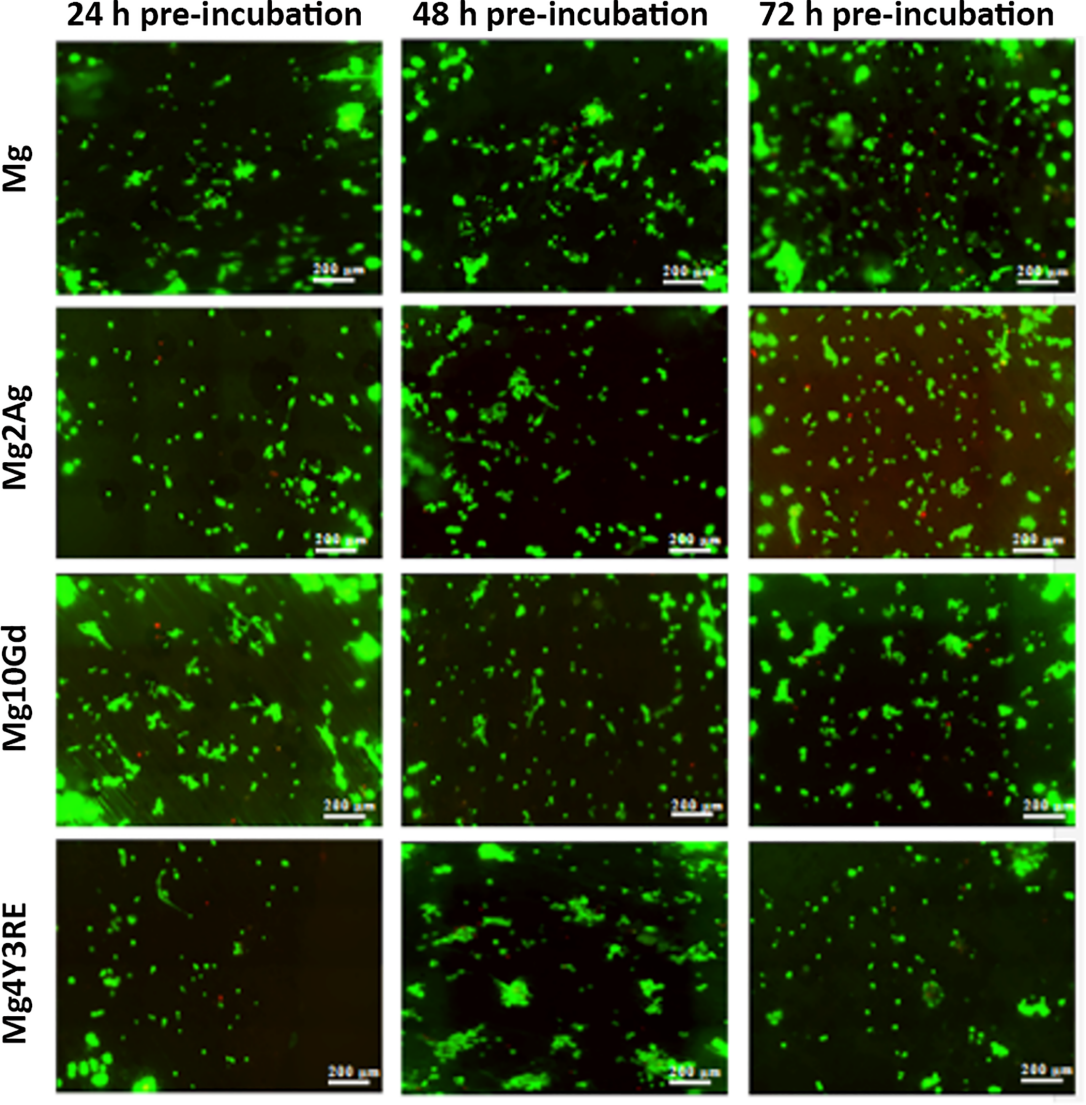
Fluorescence images of HUCPV cultured on Mg, Mg2Ag, Mg10Gd and Mg4Y3RE. Fluorescence LIVE (green)/RED (dead) staining was performed after 24 h of cell culture on 24 h, 48 h and 72 h pre-incubated samples. Monochrome images were taken in large image mode (4x4) at 20x magnification.

**Fig 5:**
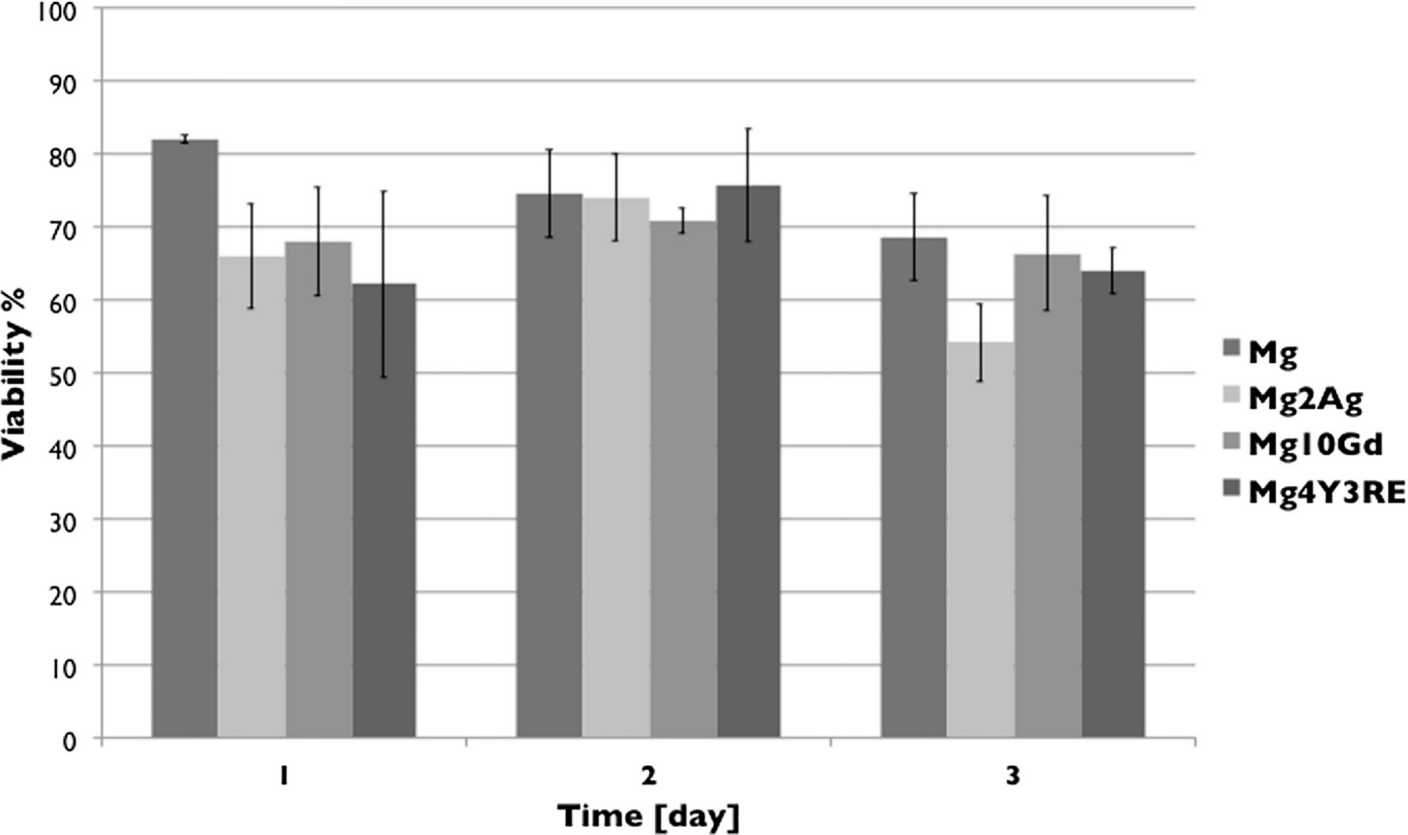
FITC intensity quantification for living HUCPV cultured for 24 h on Mg, Mg2Ag, Mg10Gd and Mg4Y3RE at 24 h, 48 h and 72 h pre-incubation states. Measurements were obtained from images taken form 3 samples of each material at each pre-incubation condition after LIVE/DEAD assay. Significance level was set at p < 0.05; n = 3 per group.

Fig 4 shows a panel of 20x magnification images obtained from LIVE/DEAD staining after 24 h HUCPV culture on each material in each pre-incubation state. The number of viable cells is observed to be comparable on each material surface. Few red cells were also detected on each sample regardless the incubation time. From the dye quantification (Fig 5), however, it is shown that cell viability slightly increased on Mg2Ag and Mg4Y3RE in their 48 h pre-incubated state, following with cell reduction at 72 h material pre-incubation. Stable cell viability was detected on Mg10Gd in different degradation states.

#### Cell actin cytoskeleton and focal adhesion on Mg-based alloys in different degradation states

Representative images of HUCPV were acquired using the multichannel filter mode in order to visualize the mapping of the three stained cell structures simultaneously.

**Fig 6:**
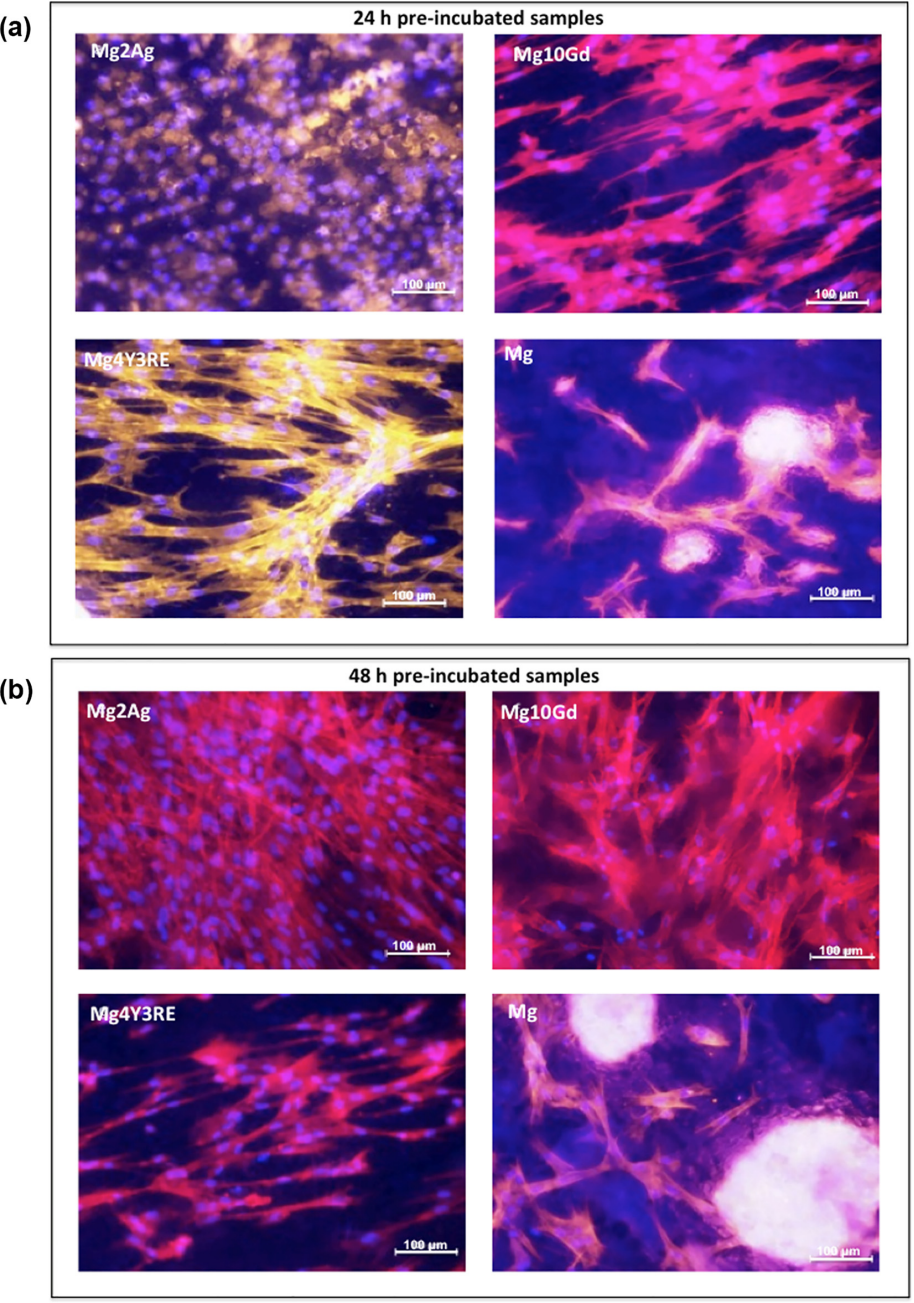
Confocal fluorescence microscopy of focal adhesion and actin cytoskeleton in HUCPV cells cultured for 24 h on Mg2Ag, Mg10Gd, Mg4Y3RE and Mg on 24 h (a) and 48 h (b) pre-incubated samples. Focal contacts were stained with vinculin monoclonal antibody (green); actin filaments were stained with a mixture of anti-mouse secondary antibody (FITC) and TRITC-conjugated phalloidin (red); nuclei were stained with DAPI (blue). Images at 20x were merged displaying the triple labeling (yellow areas obtained from the overlapping of red and green labelling).

Micrographs at 20x magnification were used to compare cell growth and adhesion structures (Fig 6) when cultured for 24 h on Mg2Ag, Mg10Gd, Mg4Y3RE and Mg in two states of degradation, after 24 h (Fig 6A) and 48 h (Fig 6B) of incubation with culture medium in controlled conditions. Actin structures seemed to be more developed on Mg2Ag after 48 h of degradation, while only weak yellow spots are visible in the corresponding 24 h pre-incubated Mg2Ag image. Even though the overall distribution did not visibly change between the two pre-incubated Mg2Ag surfaces, the counterstained nuclei in the 48 h pre-incubated micrograph seemed to be more elongated. A similar situation is observable for Mg10Gd: HUCPV covered a wider surface area when cultured on 48 h pre-incubated surface while they seemed to undergo a lasting growth on Mg10Gd in 24 h degradation state. However, in both cases, cells showed a well-defined cytoskeletal structure and ovoid nuclei. A different behaviour is observed for cells cultured on Mg4Y3RE, which appeared to undergo a decrease in cell density after a further 24 h of degradation but they were still characterized by elongated actin filament architecture. No obvious differences are visible for Mg, on which cells were clustering together.

**Fig 7:**
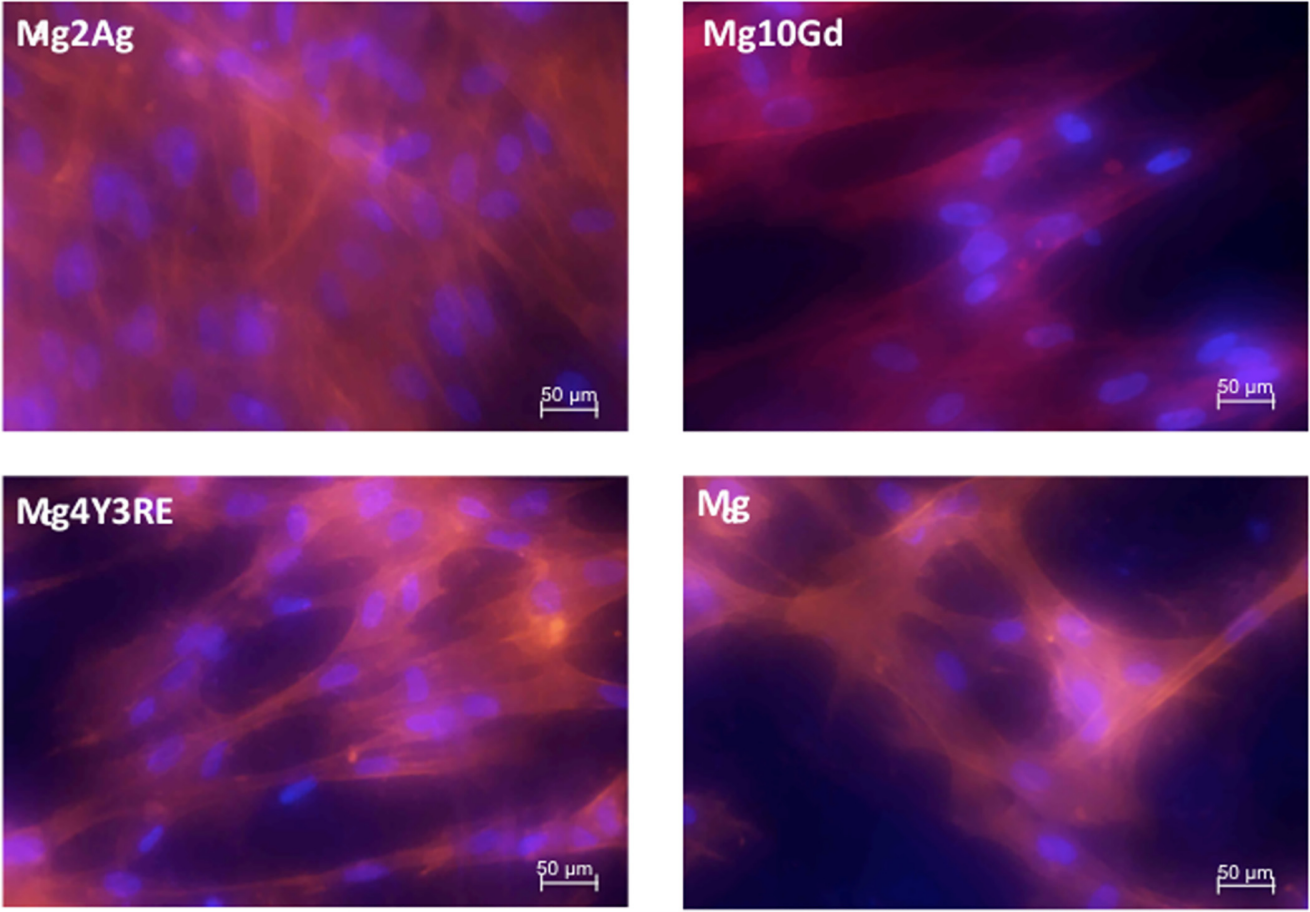
Confocal fluorescence microscopy of focal adhesion and actin cytoskeleton in HUCPV cells cultured for 24 h on Mg2Ag, Mg10Gd, Mg4Y3RE and Mg on 48 h pre-incubated. Focal contacts were stained with vinculin monoclonal antibody (green); actin filaments were stained with a mixture of anti-mouse secondary antibody (FITC) and TRITC-conjugated phalloidin (red); nuclei were stained with DAPI (blue). Images at 40x were merged displaying the triple labeling (yellow areas obtained from the overlapping of red and green labelling).

The 40x magnification images (Fig 7) gave a better observation of cellular adhesion structures and coverage on 48 h pre-incubated samples. Elongated nuclei and an actin network shaded of red and yellow are presented in all cases. HUCPV cells on Mg4Y3RE exhibited a more highly ordered actin structure, which started to be aligned with cell-to-cell connections. A similar morphology and directionality is found for HUCPV growing on Mg10Gd, whereas HUCPV on the Mg2Ag showed non-homogenous distribution. It is also notable that focal adhesion points are rather more visible when observing at Mg4Y3RE. They corresponded to two strong yellow areas given by the overlap of the red actin staining and the green focal contact staining.

## Discussion

Large amounts of hydrogen generated from the degradation mechanism of Mg4Y3RE materials results in gas bubble accumulation inside tissues, loosening the biomechanical interlocking at the bone-implant interface [31]. For this reason, other alloying elements have been combined with magnesium to modulate degradation properties and mechanical strength in order to temporarily support the healing of the injured bone tissue and allow proper initial cellular growth [32]. The most recently examined Mg alloys are magnesium-silver (Mg-Ag) and other magnesium-rare-earth (Mg-RE) alloys [33–35]. In the present study, different magnesium alloys with controlled degradation rates and defined alloying components were tested aiming at biodegradable bone applications. Therefore, it was of significant interest to investigate the degradation profile under specific cell culture conditions and how it affects the early response of human umbilical cord perivascular stem cells in terms of cell viability, spreading and adhesion structures.

When testing the biodegradation properties of magnesium-based materials in cell culture medium the presence of hydrogen carbonate, as an important buffering component, can raise the possibility of MgCO_3_ formation according to the total reaction:

$$\mathrm{Mg}+{\mathrm{HCO}}_{3(\mathrm{eq})}^{-}+H_{2}O_{\left(l\right)}\to {\mathrm{MgCO}}_{3(s)}+H_{2(g)}+{\mathrm{OH}}_{\left(\mathrm{aq}\right)}^{-}$$ (2)

It has been reported that Mg-CO_3_ phase formed on Mg when samples were immersed in a cell growth media supplemented with FBS [9]. Furthermore, calcium and phosphate proved to be other important components in the cell culture media, as their presence can alter the degradation profile by inducing Ca-P-Mg phases precipitation [36]. In our study, EDX area mea show that the chemical composition of the degraded surfaces differs between the different alloys. Mg10Gd and Mg4Y3RE show comparable elements compositions, whilst Mg2Ag exhibit high P content that may correspond to Ca-P phase precipitation, which tend to form a homogenous protecting layer [37].

Overall, the biological observations showed that cells differently adhered on all the three alloys as well as on Mg *in vitro*. Initial attachment of stem cells to the implant surface has been shown to determine cell fate and function, driving proliferation and differentiation into specific cell phenotype. In order to provide a comprehensive preliminary overview of cell-substrate interactions, material cytotoxicity assay and a triple staining immunofluorescence analysis of cells cultured for 24 h were performed. In general, good density of living cells was observed for all materials in each corroded state. HUCPV cells viability was lower that 80% on each alloy in each pre-incubated state and this is likely correlated to the cell culture modalities in direct contact with highly reactive Mg samples. However, cell reduction was not significantly lower than cell viability on the experimental control (Mg) at 24 h pre-incubation time. This is a clear indication that these materials exhibit no cytotoxic effect on HUCPV. Mg10Gd seemed to maintain the level of cell viability as the pre-incubation time increased, with % close to 70. From the immunocytochemical staining, homogeneous cell growth was observed for magnesium alloys, whereas the cells growing on Mg presented a tendency to grow in clusters. This could be correlated to the morphology of the degradation structures on high pure magnesium samples, particularly after three days of immersion.

The assembly and organization of actin filaments depend on the degree of cell spreading. The directionality of filopodia extension depending on the microenvironment and substrate conditions may be determinant of the mode of spreading. Cells have been shown to spread either isotropically (equally in all directions) or anisotropically, where the cells develop extensions unevenly in multiple directions [13, 38]. When observing the Mg2Ag alloy, it was evident that cell adhesion and distribution did not morphologically change as the pre-incubation time increased. Even though the cell density did not decrease between the 24 h- and 48 h pre-incubated samples, actin structures were less developed or not even visible compared to the other alloys. A similar trend in cellular density was observed on the Mg10Gd corroded surfaces at both 24 h and 48 h, but with a well-defined and developed cytoskeletal structure compared to the Mg2Ag. Mg4Y3RE presented reduced cell density as a function of surface degradation. From these observations, it can be suggested that Mg10Gd seemed to possess better capability to induce filopodia and lamellopodia development with a stable cell layer compare to Mg2Ag.

The reduction in cell density on the Mg4Y3RE samples can be explained by its higher degradation rate compared to the Mg2Ag and the Mg10Gd. This is in accordance with the *in vitro* degradation analysis which showed that pH and osmolality of the medium increased with 72 h of immersion time. It has been demonstrated that hypertonic medium (medium with a higher solute concentration than intracellular environment) represents a stressful condition for cells and it induces a cell growth depression [39–41]. In hypertonic extracellular microenvironment, water diffuses out of the cells due to osmosis, leading to cell shrinkage. Therefore, cell shrinking may be one of the reasons of the cell reduction on the Mg4Y3RE surface.

The cell spreading results suggest that there could be also a rapid cell response to changes in the pH of the medium. Also the pH alteration may contribute to influence cell spreading. This is in accordance with many biological studies that have stated an inverse correlation between pH and cell growth [42–44]. Cells would likely be exposed to an increase in pH for a short period of time when seeded on biodegradable Mg alloys [5, 9, 15, 16, 45]. It is notable that the Mg4Y3RE induced a higher pH increase in the medium due to its more rapid degradation rate and this could be considered as another relevant cause of reduced cell density in this material. Particularly, focal adhesions are pH sensitive and these structures are proven to be more stable in an environment with values below pH 6.0 and above 7.2 [46, 47]. Thus, it can be said that, under physiological conditions, a strong alkalinisation of the microenvironment given by material degradation can be counteracted by the extracellular pH that becomes acid in physiological context such as wound healing and skeletal regeneration [48]. This buffering effect could then preserve a weak but still alkaline environment, which promotes the development of adhesion structure.

When observing the surface topographical degradation, it was shown that Mg2Ag presented a levelling of the surface texture (edges) between 24 h and 48 h significantly reducing the surface area in Mg2Ag, followed by a significant increase in the surface roughness after 48 h of immersion for all materials. This may be due to a higher susceptibility for formation of small and localized degradation areas, which started aggressive especially for Mg2Ag. This strong alteration in the surface topography of Mg2Ag may be one of the reasons for the weakness in cellular adhesion onto this material. Conversely, the surface degradation appeared to be more homogeneous along the incubation time for Mg4Y3RE and Mg10Gd, which suggests that these alloy are slightly less sensitive to the “pitting-like” attack, permitting the correct cell initial recruitment and adhesion when placed in living tissues.

Therefore, the *in vitro* results of the current study showed that HUCPV adhesion and spreading were likely reduced on the Mg4Y3RE alloy due to the increased pH and osmolality (linked to material’s higher degradation rate). On the other hand, even though cell density seemed to be preserved due to the lower degradation and alkalinization, cells grown on Mg2Ag exhibited less focal adhesion development compared to Mg10Gd and Mg4Y3RE.

Several reviews on biodegradable magnesium based alloys have explained that a moderate and homogeneous degradation is required while the bone healing process occurred *in vivo* [49–51]. Therefore, it can be suggested that Mg10Gd alloy may represent a suitable alternative to the commercially available Mg4Y3RE alloys, since it possessed a reduced and more constant initial degradation that allows undifferentiated cells to properly adhere and spread on it.

## Conclusion

No adverse cell reaction was observed on all materials in each degradation state. Mg4Y3RE presented the highest degradation rate and, therefore, the highest trend in pH and osmolality. HUCPV were likely reduced when grown on this alloy. However, the cellular structures appeared better developed on Mg4Y3RE and Mg10Gd compared to Mg2Ag.

This work showed how the degradation parameters, the surface topography and chemistry of the magnesium-based materials influence the initial cellular adhesion and spreading. It is suggested that it may be worth testing the cellular and tissue response to other Mg alloys with different percentages of Gadolinium as suitable alloying element.
